# Microwire vs. Micro-Ribbon Magnetoelastic Sensors for Vibration-Based Structural Health Monitoring of Rectangular Concrete Beams

**DOI:** 10.3390/s25123590

**Published:** 2025-06-07

**Authors:** Christos I. Tapeinos, Dimitris Kouzoudis, Kostantis Varvatsoulis, Manuel Vázquez, Georgios Samourgkanidis

**Affiliations:** 1 Department of Chemical Engineering, University of Patras, 26504 Patras, Greece; msci1481@upnet.gr (C.I.T.); phy5255@upnet.gr (K.V.); 2 Institute of Materials Science of Madrid, ICMM/CSIC, 28049 Madrid, Spain; mvazquez@icmm.csic.es; 3 Department of Mechanical and Manufacturing Engineering, University of Cyprus, 1678 Nicosia, Cyprus; g.samourgkanidis@gmail.com

**Keywords:** magnetoelastic materials, microwires, vibration sensors, concrete structures, damage detection

## Abstract

Two different magnetoelastic Metglas materials with distinct shapes were compared as sensing elements for the structural health monitoring of concrete beams. One had a ribbon shape, while the other had a microwire shape. The sensing elements were attached to different concrete beams, and a crack was introduced into each beam. The beams were subjected to flexural vibrations, and their deformations were recorded wirelessly by coils, detecting the magnetic signals emitted due to the magnetoelastic nature of the sensors. Fast Fourier Analysis of the received signal revealed the bending mode frequencies of the beams, which serve as a “signature” of their structural health. In these spectra, the ribbon-shaped sensor exhibited a 1.4-times stronger signal than the microwire sensor. However, the extracted mode frequencies were nearly identical, with differences of less than 1% both before and after damage. This indicates that both sensors can be used equivalently to monitor structural damage in concrete beams. The damage-related relative frequency shifts ranged from −0.01 to −0.03, with similar results for both sensors. Thermal annealing was also studied and appeared to significantly enhance the signal by 10–30%, likely due to the relaxation of internal stresses induced during the rapid solidification synthesis of these materials. This enhancement was more pronounced in the ribbon-shaped sensor. This study is the first to utilize a magnetoelastic microwire sensor for damage detection in concrete beams.

## 1. Introduction

Mechanical structures are often exposed to adverse conditions that impact their performance, with most of the damage arising from the formation and spread of cracks in the materials. Detecting and characterizing these faults is essential to prevent material failure and ensure a smooth operation. Two critical research areas focusing on these issues are Non-Destructive Evaluation (NDE) and structural health monitoring (SHM). SHM, in particular, plays a vital role in maintaining the safety and operational efficiency of structures. Unanticipated failures can severely affect human safety and daily productivity. Damage typically begins as microscopic fatigue cracks within the material, which grow over time and compromise the structure’s integrity. Detecting these micro-cracks is challenging and often requires costly and intrusive methods, like electron microscopy or ultrasound, which involve extracting parts of the structure for inspection. However, several affordable and non-intrusive techniques have been developed to detect sub-millimeter cracks on-site, allowing for early detection and effective SHM.

The most frequently employed SHM methods involve fiber optics [1] and acoustic sensors for acoustic emission and wave propagation control [2], and accelerometers [3]. Emerging techniques feature Micro-Electromechanical Systems (MEMSs) [4], piezoelectric paints [5], Large Area Electronics (LAEs) [6], and non-contact laser methods [7]. Additionally, various algorithmic data analysis techniques are used in conjunction with these sensor methods to detect and characterize cracks [8]. In general, vibration-based structural health monitoring (SHM) methods are widely embraced. Avci et al. [9] reviewed the evolution of vibration-based structural damage detection, highlighting the shift from traditional methods to advanced ML and DL techniques. They emphasized the advantages of compact 1D CNNs, which enable accurate damage detection from raw acceleration data without manual feature extraction. This study also addressed key challenges, such as the reliance on labeled data, and proposed solutions, like simulated training data and unsupervised learning, to enhance real-world applicability. This method aims to achieve several objectives: detecting structural damage, pinpointing its location and severity, assessing structural safety, predicting the remaining service life, and determining optimal maintenance strategies, where feasible. The vibration properties of a structure depend on its physical parameters [10]. When structural damage occurs, these parameters change, thereby serving as indicators of the structure’s health. By monitoring signals from sensors installed on the structure, vibration characteristics can be extracted and modal analysis can be used to assess its health condition [11].

A very common sensing technology for vibration detection is the use of accelerometers because they are very versatile for various applications and can be easily integrated with MEMS devices. Perhaps the only disadvantage is the lack of sensitivity at low Hz frequencies, which are the typical mechanical vibration frequencies for large structures. Additionally, they cannot be integrated inside the beam material but only placed on the outside, giving the microwire sensing material of the current work a clear advantage. Examples of using accelerometers as vibration sensors include ref. [12], where the authors combine frequency response data with artificial neural networks to detect and prognose multi-fault detections in pipelines, and ref. [13], where frequency response data from small-scale wind turbines are filtered in order to obtain better signals for blade damage. Damage detection in steel alloy structures is detected by frequency responses in ref. [14], and in ref. [15] a hammer impact test is performed on cracked and no-crack beams made of steel to obtain the change in natural frequencies and mode shapes. Nanocomposites are integrated inside cement beams in ref. [16] in order to create a smart material, which delivers vibration measurements via resistivity tests, and the results are in good agreement with the accelerometry tests. Finally, in ref. [17], a two-step procedure is developed for damage identification in beam structures based on changes in the modal curvature.

Magnetoelastic materials are among the vibration sensor materials that have not been extensively researched until recently. These materials have demonstrated high effectiveness in sensing vibrations due to their unique capability to transform mechanical strains into detectable changes in magnetic properties. This transformation, characterized by a high conversion ratio between elastic energy and magnetic energy, is known as the “coupling coefficient” (k). A specific category of these materials, known as “Metglas”, stands out for its metallic amorphous nature and impressive conversion ratios (0.70 < k < 0.97). Modzelewski et al. [18] measured the magnetomechanical properties of Metglas 2605CO and 2605SC under various annealing conditions, reporting maximum coupling factors of 0.71 for 2605CO and significantly higher values (0.9–0.96) for 2605SC due to its lower anisotropy, along with low losses and predominantly rotational magnetization. Supporting this, Savage and Spano [19] demonstrated that Metglas 2605SC had superior magnetomechanical coupling, driven by its well-defined anisotropy and large ∆E effect. They predicted strain gages using this material could be 10^3^ to 10^5^ times more sensitive than conventional ones, with low noise and a strong low-frequency performance. This makes Metglas particularly well-suited for sensing applications. The system functioned both as a working electrode for electrochemical sensing and as a magnetoelastic sensor. It showed a strong electrocatalytic response, rapid detection (30 s), and high sensitivity with a detection limit of 0.16 µM, demonstrating its stable, reproducible, and selective performance. Also, Song et al. [20] developed highly compressible, self-powered, 3D-printed magnetoelastic sensors with tunable mechanical properties and wide strain ranges. Fabricated using sacrificial molds and magnetic composites, these sensors were integrated into robots for improved control and interaction recognition. Their design overcomes prior limitations by offering high compressibility, a broad dynamic range, and efficient 3D integration, advancing human–machine interfaces and expanding soft sensor applications. Similarly, Shekhar et al. [21] developed a wireless sensor system using commercially available Metglas 2826MB to monitor mammalian cell attachments in 2D cultures, demonstrating a linear correlation with fibroblast growth and providing the real-time monitoring of cell attachment and proliferation, highlighting its potential for non-invasive biological sensing. Typically composed of thin, metallic, amorphous ribbons around 25 µm thick, Metglas has proven its efficacy in sensing vibrations. In our previous study [22], we investigated and characterized Metglas 2826MB3 ribbon as a vibration sensor. This material offers several benefits, including low cost, the ability to function without sophisticated and expensive electronic systems (operating at low frequencies in the kHz range), and a contactless nature that eliminates the need for electrical connections, relying solely on magnetic properties for signal detection.

Another class of materials suitable for use as vibration sensors due to their magnetoelastic behavior are magnetic microwires [23], which constitute the primary focus of this research. These microwires consist of a cylindrical ferromagnetic core with micrometer-scale diameters. Magnetic microwires are fabricated by an ultrarapid solidification technique referred to as the in-water-quenching method. This ultrarapid cooling creates the desired amorphous structure essential for the soft microwire’s magnetic properties. Its composition, similar to the case of amorphous ribbons, consists of ferromagnetic elements, like iron, cobalt, or nickel, and amorphizing metalloids (e.g., Si, B, etc.) to result in an amorphous alloy. The water-quenched microwires have a larger cross-section than other families of microwires (e.g., glass-coated microwires) [24] and compared to them, they inhibit a larger magnetic response. They have been employed as sensing elements in various sensor devices (e.g., as orthogonal fluxgate or giant magnetoimpedance sensors). Panina et al. [25] investigate the microwave magnetic properties of soft magnetic microwires and their composites, demonstrating wideband dynamic permeability and tunable microwave impedance. These properties enable applications in wireless sensors, artificial magnetic dielectrics, and metamaterials with negative or tunable refractive indices. Composites with circumferential anisotropy show significant effective permeability at low wire concentrations, paving the way for advanced tunable microwave devices. Additional treatments, such as annealing, may be utilized to improve the microwire’s magnetoelastic properties [26], a technique also employed in this research.

This study builds upon our previous research [27], which involved detecting damage in rectangular concrete beams by analyzing vibrations and performing frequency modal analysis with Metglas ribbons as sensors. In the current work, we enhance this technique by utilizing magnetic microwires as sensing elements on concrete beams and comparing their performance to that of Metglas ribbons. Our ambition is to embed the microwires inside the concrete material, thus making a smart composite, with the microwires playing the role of the reinforcement material and the role of the sensing material at the same time. We have performed this experiment in the past with Metglas ribbons and plastic beams, and the preliminary results show that it can be conducted with concrete beams too. The first step toward this innovation is to compare the two sensing materials on the external surface of the concrete beams, before we embed them inside the material, as the signal weakens in the latter case.

## 2. Materials and Methods

### 2.1. Sensing Elements

As was mentioned in the Section 1, two types of sensors were used in the current work to detect the vibrational modes of concrete beams, both of which were made of amorphous magneto-elastic material but with different shapes: (a) a 30 μm-thick ribbon and (b) and a 100 μm-diameter microwire. The most important property of magneto-elastic materials is that their magnetization varies upon the application of an external stress on them. The magnetization can be easily detected in a contactless fashion by a close coil. This property, together with the micrometric dimension of these materials, makes them ideal candidates for vibration sensors when they are attached to a vibrating structure, as the structure excites them and the coil can detect the vibrational spectrum, as it will be shown below.

The magnetoelastic ribbon (indicated as “RB” hereafter) is a commercial material known as “Metglas 2826MB3”, manufactured by Metglas Inc. (Conway, SC, USA), which is an amorphous metallic alloy with an average composition of Fe_40_Ni_38_B_18_Mo_4_. Concerning its magnetic properties, it is a soft material that together with its small thickness of 25 μm, makes it ideal for transformers and sensing applications.

**Figure 1:**
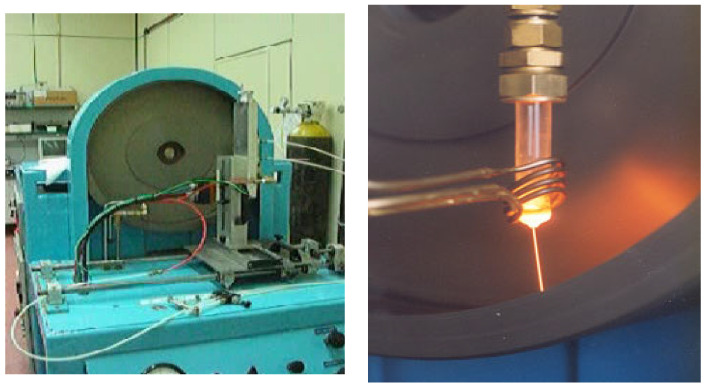
(**Left**) In-rotating-water cast unit and (**Right**) image taken during the fabrication of the magnetic microwire. The molten alloy inside the quartz tube was plunged into the water, which was set in a rotating flow inside the drum.

On the other hand, the microwire (indicated as “MW” hereafter) was fabricated in the laboratories of ICMM/CSIC having an Fe_75_Si_10_B_15_ alloy composition, and a high positive-saturation magnetostriction of 32 ppm. This microwire shows, like the ribbon, a soft magnetic character, and the main difference is its cylindrical shape. The microwire is expected to result in a lower sensor response than the ribbon owing to its smaller cross-section. However, it can be used both as a fiber reinforcement and sensing material, a combination which makes it a smart material. The microwire with the around 100 micrometer diameter was fabricated by the in-rotating-water technique, where the desired precursor polycrystalline alloy was molten inside a quartz tube because of high-frequency induction at 1250 °C, as shown in Figure 1. The end of the quartz tube had a small hole through which the molten alloy was ejected under the action of argon overpressure and plunged into cold rotating water. The high cooling rate to room temperature was achieved in a few milliseconds, which resulted in an amorphous alloy with a cylindrical shape. Note that, in the case of the amorphous ribbon, the process was similar, where the alloy was quenched by its ejection onto the surface of a CuBe rotating wheel.

### 2.2. Sample Preparation

**Figure 2:**
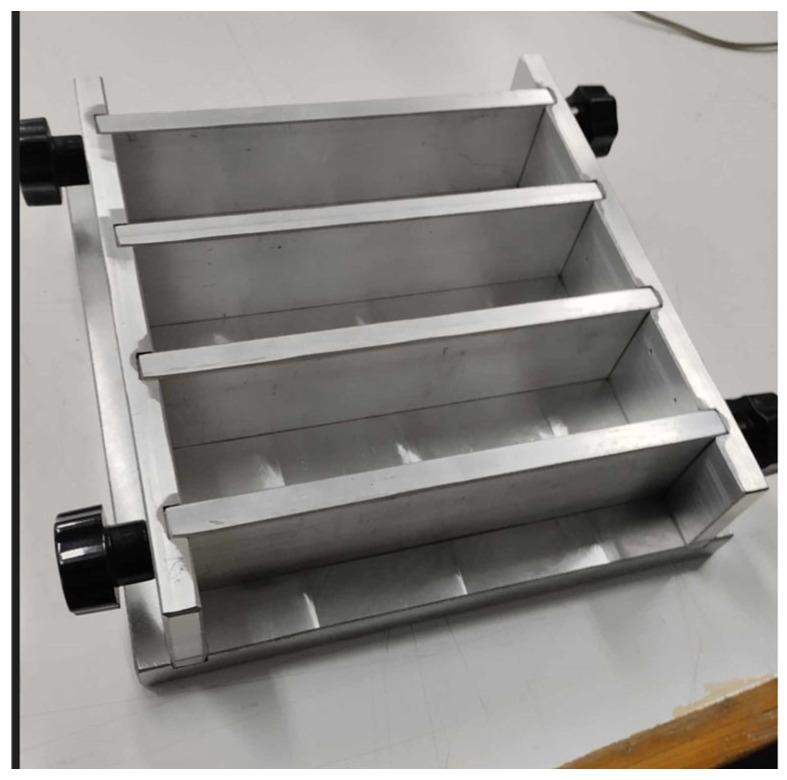
Molds for making concrete beams.

Standard concrete beams were used in this study, which were fabricated in our laboratory from the following raw materials: (a) a Durostick, commercial, gray, Portland cement-type PlusCem 52.5N, which is a commercial fiber-reinforced hydraulic binder, (b) mechanical oil, (c) petrolatum, (d) distilled water, and (e) standard sand with SiO_2_ aggregates > 99%. The percentage by weight of water/cement/sand was equal to 1:2:6. The concrete samples were manufactured according to the EN 196-1 standard [28], using a simple mixer and the aluminum molds shown in Figure 2 with internal dimensions of 4 × 4 × 16 cm^3^. The molds were oiled for easy demolding and the joints were sealed with petroleum jelly. After pouring the mix into the molds, it was solidified by placing the molds in a refrigerator for one day. Following this, the samples were demolded and immersed in distilled water for 27 days to harden.

## 3. Experimental Methods

### 3.1. Experimental Setup for Exciting the Beams

**Figure 3:**
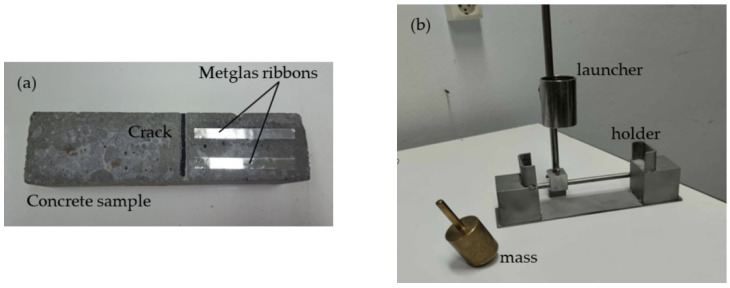
(**a**) Concrete sample with double sensors attached on it, and (**b**) sample holder base and excitation mechanism.

Shown in Figure 3a is a typical 4 × 4 × 16 cm^3^ concrete beam sample on which two Metglas sensor ribbons (RBs) were glued using commercial double-sided tape. The tape was used with success in [27] and it had a minimal effect on the detected signal. The ribbon sizes were 6 cm × 7 mm × 25 μm each and they covered less than half of the beam’s top surface lengthwise. A crack was introduced to the center of the beam, as shown in the photo, as this position produced the best signal compared to all other positions lengthwise. The beams needed to be excited to bending vibration and this was performed with the mechanism in Figure 3b, which is composed of a holder, where the beam is placed horizontally, and a heavy mass in the form of a pointer, which is released from a fixed height from a cylindrical launcher. The mass landed on the left side of the beam (in Figure 3a) to avoid damage to the sensors. The gravitational energy of the mass at the launcher was converted to vibrational energy, once the pointer impinged on the beam. The pointer had a smooth end to avoid concrete failure during impact. Similarly to the ribbons, the microwire sensors were attached to the beam with double-sided tape.

### 3.2. Sensor Signal Comparison

**Figure 4:**
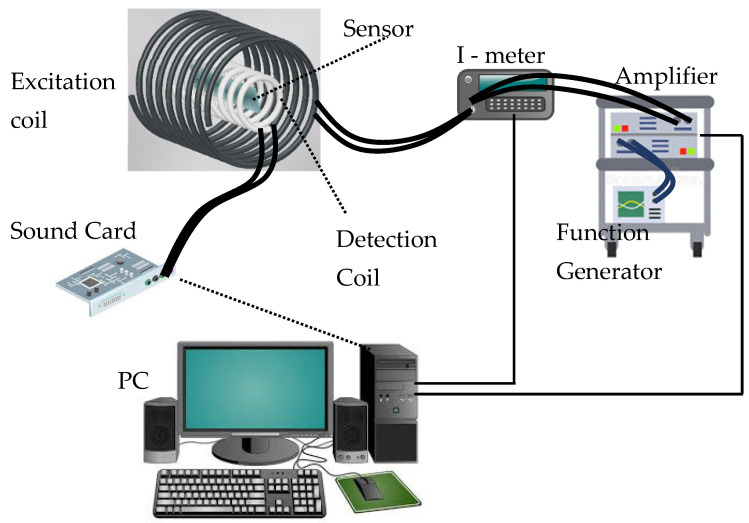
Sensor signal comparison setup.

Before any measurement was made, the two sensing elements needed to be characterized and their signals compared. As these sensors are magnetic, an easy characterization method is to insert them inside a detection coil 4cm long, as depicted in Figure 4, and apply an external AC magnetic field with the help of an excitation coil 6 cm long. The magnetoelastic nature of these materials caused them to vibrate at the same frequency as the field and emit an alternating flux inducing a corresponding AC emf voltage to the detection coil. This voltage was collected by a sound card (analog input) with the help of a personal computer (PC), and this emf is basically the measured sensor signal in Volts. The PC controlled the amplifier gain to deliver a constant current to the excitation coil and thus to generate a constant-amplitude AC field.

**Figure 5:**
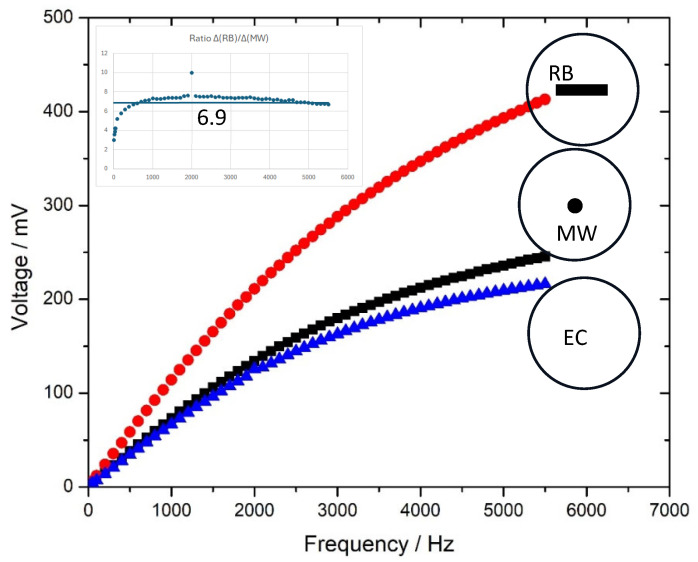
Plots of sensor signals received from the setup of Figure 3, Empty coil (EC blue), Microwire (MW black) and Ribbon (RB red). The circles represent the detection coil cross section with the sensor inside it. The inset shows the ratio of the two sensor signals after the empty coil signal is subtracted.

The resulting measurements for the two sensors, RB and MW, both 6 cm in length, are shown in Figure 5. For comparison, the signal of the empty coil (EC) is plotted along with the signal of the two sensors. Clearly, the ribbon signal (RB) is higher than the corresponding microwire signal (MW) and, as expected, both are higher than the empty coil signal. Shown also schematically in the figure to the right are the cross-sections of the detection coil for the three cases. To compare the signal from the two sensors, we took the ratio Δ(RB)/Δ(MW) of the two signals after the empty coil signal was subtracted, i.e., Δ(RB) = RB − EC and Δ(MW) = MW − EC, and this ratio is plotted versus the frequency in the small inset of Figure 5. Excluding the low-frequency regime, this ratio is almost constant, with an average value of 6.9. The reason that the two sensor signals are different is mainly due to two causes: (a) the differences of the cross-section of the two sensors, as is apparent in the schematic drawings to the right of Figure 5, and (b) the differences in the magnetic susceptibility of the two materials. Relevant calculations will be provided in the next section, where the magnetic measurements are presented.

### 3.3. Magnetic Characterization of Sensors

**Figure 6:**
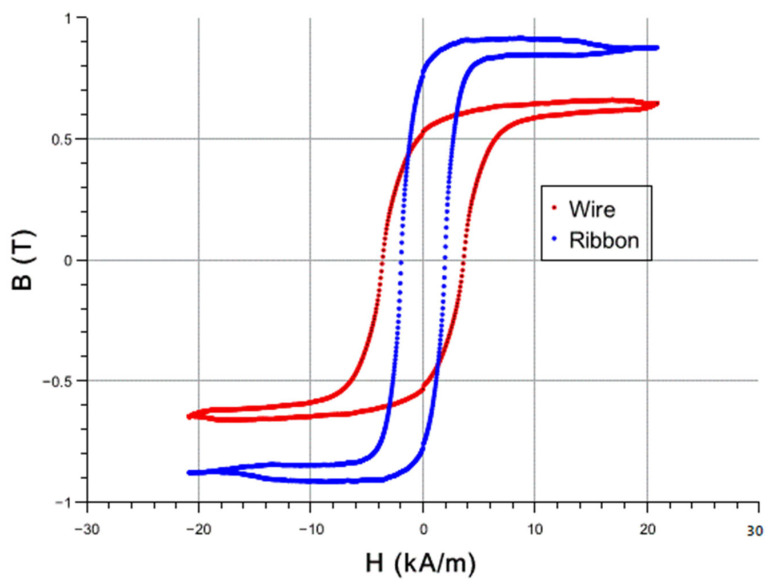
Magnetic hysteresis loops of the two sensors: RB and MW.

Shown in Figure 6 are the magnetic hysteresis loops for the two sensors, both with a length of 6 cm. Unfortunately, the two loops were received via different techniques and are plotted on the same graph only for the sake of comparison. The ribbon data were collected by an AC susceptibility technique at a frequency of 1000 Hz. The ribbon loop looks like it reached its saturation, which is 0.9 T according to the literature. On the contrary, the microwire loop still has a small slope, and higher fields are required to bring it to saturation, which is typically close to 1.6 T for this synthesis.

Similar to Figure 5, the signal differences in Figure 6 for the two sensors, RB and MW, and more specifically the slopes of the two square loops, which is known as susceptibility χ, depend on the sensor cross-sections but also on the different demagnetizing factors, due to the different shapes. We estimated the slopes of these loops to be approximately 0.715 Tm/A for RB and 0.055 Tm/A for MW, and thus the corresponding ratio is approximately 13. This is almost twice the corresponding signal ratio of that in Figure 5, which was found to be close to 7. Just to have an idea of the area contribution to this ratio, the ribbon cross-section dimensions are 25 μm thickness × 1/4 inch width, and the wire diameter is 100 μm, which leads to a cross-section ratio of about 20. Qualitatively this means that the wire has a smaller demagnetizing factor, which is expected for a cylindrical shape compared to an orthogonal shape as this factor is proportional to the length-to-width ratio. Shown in Table 1 below are the magnetic properties of the two sensing materials, converted to the same A/m units (note: 1 T = 79.5 × 10^4^ A/m), which make the susceptibility χ a unitless quantity. The table also shows the literature values for Saturation Magnetization for the sake of comparison.

### 3.4. Thermal Treatment of Sensors

The production of Metglas materials involves rapid cooling to make these materials amorphous so as to have unique properties. As a result, internal mechanical stresses develop during the process due to the non-uniform thermal contraction within the material. These mechanical stresses affect the sensor by reducing the intensity of the signal that it can produce. A way to eliminate these mechanical stresses is thermal annealing. The annealing conditions of 350 °C at 35 min were applied to the ribbon and 325 °C at 45 min were applied to the wire. It should be noted that special attention should be paid to the annealing temperature so as not to exceed the Curie temperature of these materials, which is slightly higher than the above values.

**Figure 7:**
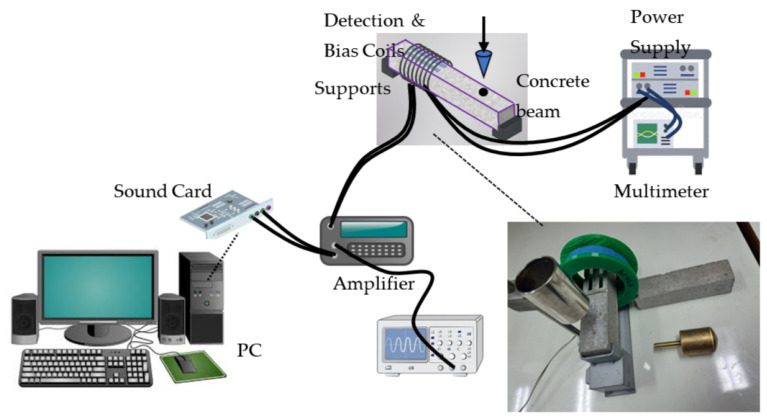
Experimental setup used to detect damage.

**Figure 8:**
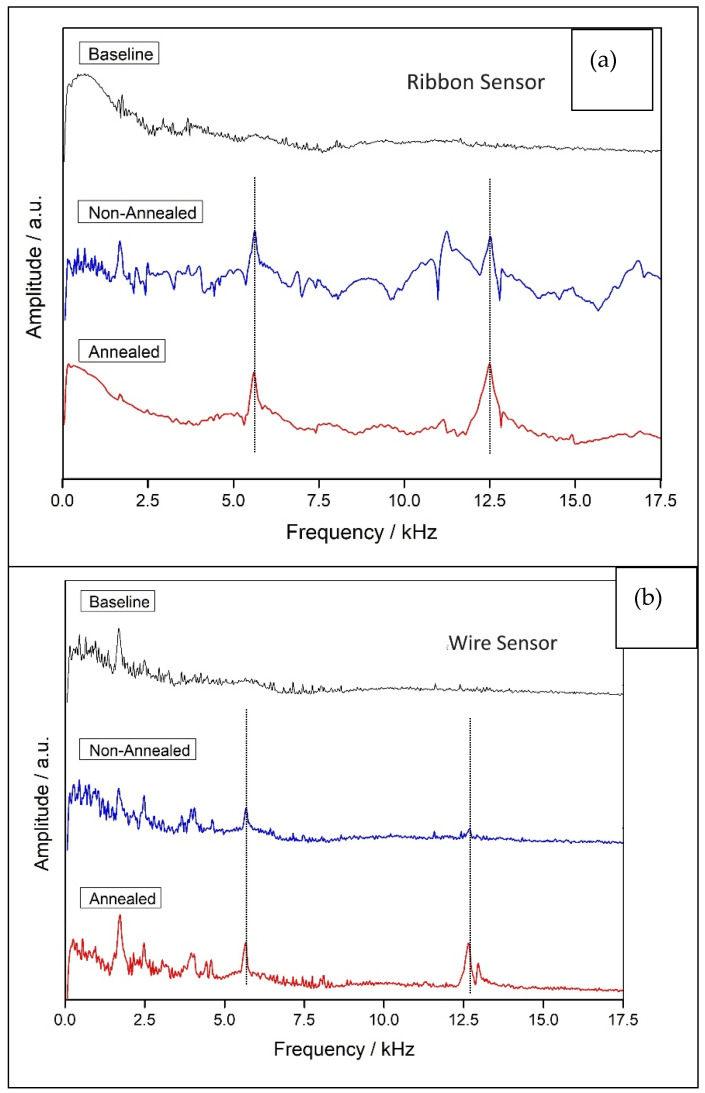
Comparison of annealed versus non-annealed sensor signal (**a**) for the ribbon and (**b**) for the microwire.

To test the effect of treatment on sensor properties, we measured the flexural vibration modes of concrete beams with and without annealing. The damage detection setup shown in Figure 7 (and the photo inset therein), was used to obtain the flexural modes of the concrete beams. In short, a concrete beam with sensors attached to it, like the one in Figure 3a, was inserted into the detection coil presented in Figure 7 and excited by the excitation mechanism shown in Figure 3b. The forced vibrations detected by the coil were collected by a PC through a sound card and Fast Fourier Transform (FFT) was applied to the data. The bending modes are then evident as dominant peaks in the FFT spectrum, as shown in Figure 8 for the two different sensors (Figure 8a for the ribbon and Figure 8b for the wire). In these figures, the baseline (black curve) corresponds to the absence of the concrete beam so as to obtain the signal resulting from the detection coil alone. The other two plots correspond to the signal from the non-annealed and annealed sensors (blue and red curves, correspondingly). In these spectra, it is evident that there are two dominant peaks present at frequencies above 5 kHz (shown by vertical, dotted lines), and it is also clear that the annealing process reinforces their amplitude, which is expected. Note for example that the second peak in Figure 8b is barely visible before the annealing process, while it is a dominant peak after annealing. This concludes that, as in the case of the ribbon sensor, annealing is necessary as a preprocessing step for the wire sensor as well. 

Table 2 shows the percentage changes in peak heights and frequencies with and without annealing for the two sensors. From this table, it is evident that the annealing process had literally no effect on the mode frequencies of the beams (changes less than 1%), which was expected and which proves that these frequencies are characteristic of the beams and not some sensor property (the same beam was used before and after the annealing process so as to have the same comparison standards). Concerning the amplitude of the peaks, there was a significant signal gain in the range from 10 to 30%, with the signal gain for the first peak being greater for both sensors (RB and MW). Finally, the gain was greater for the RB sensor than the MW sensor for both peaks. Perhaps this is due to the greater symmetry of the cylindrical shape of the wire sensor compared to the rectangular shape of the ribbon, which results in less local remaining stresses during the rapid-cooling production process.

## 4. Results and Discussion

### 4.1. Comparison of the Two Sensors for Crack Detection Experiments

**Figure 9:**
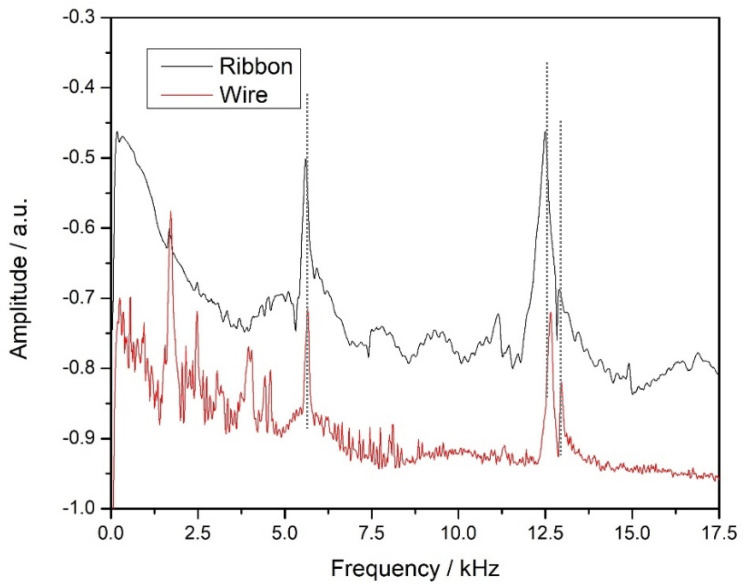
Comparison of the FFT spectra of the two sensors received from the same concrete beam in the setup of Figure 7.

The two sensors (RB and MW) were attached to different but similar concrete beams, and detection experiments were performed using the experimental setup of Figure 7, as described in the previous section. The received FFT results are shown in Figure 9. From these spectra, we can see that the ribbon sensor produces a stronger signal than the wire, which is expected according to the discussion in Section 3.2. Table 3 shows the frequencies of the three main peaks of the two sensors, and it can be seen that there is no significant shift of the peaks from one sensor to the other, which is expected.

**Figure 10:**
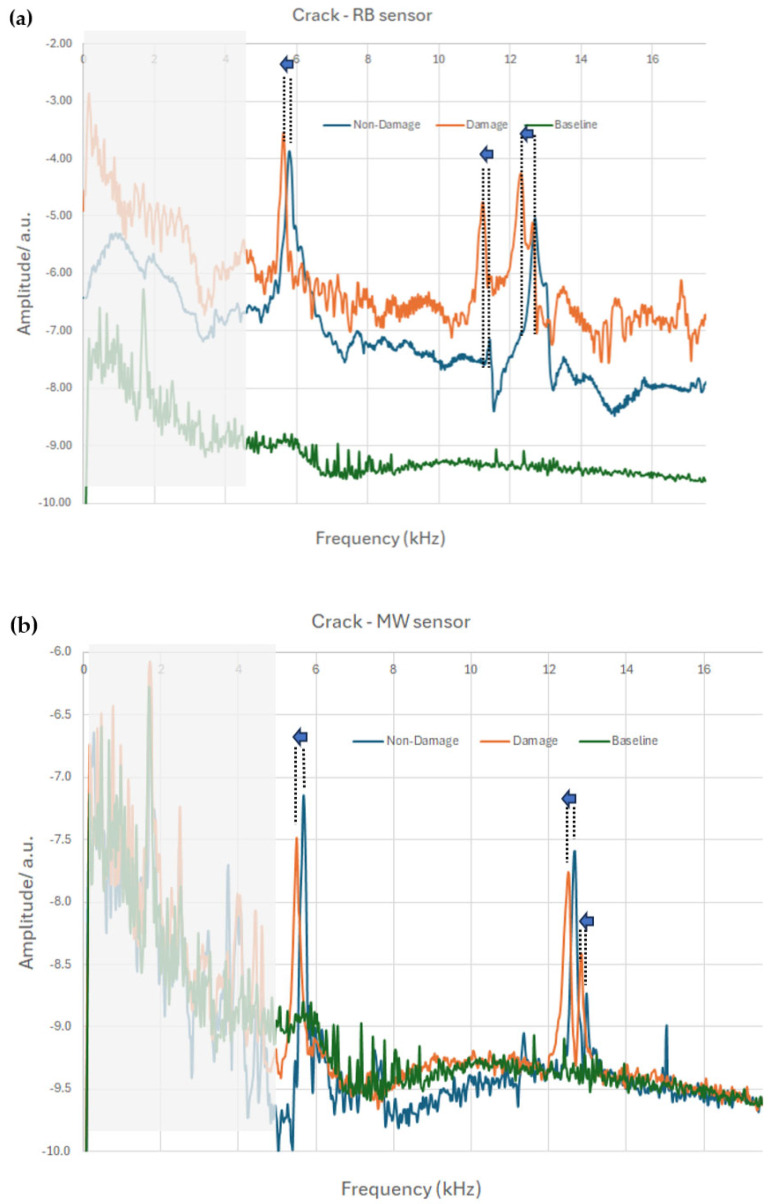
Comparison of the FFT spectra of the two sensors, (**a**) RB and (**b**) MW, when a crack is introduced in the same concrete beam (per sensor) in the setup of Figure 7.

Shown in Table 3 are the frequencies of the three main peaks of the two sensors (a single peak around 5 kHz and a double peak around 12.5 kHz), and it can be said that the differences between the two sensors are insignificant, less than 1.5%, which is of the order of the experimental error. On the other hand, the signal amplitude seems to be different, something which is expected according to the discussion in Section 3.2. An estimate for this difference can be made in Figure 10 by taking the height difference of the first peak with respect to the background noise around it. For example, for the ribbon sensor, the peak is around −0.5 in the arbitrary units (a.u.) shown, while the background is estimated to be around −0.75 a.u. (close to the peak), and thus the clear peak height is their difference of 0.25 a.u. The same estimation for the wire ribbon leads to a peak value of −0.72 a.u. in the graph with a corresponding background of around −0.9 a.u., which produces a clear value of 0.18 a.u., and thus the signal ratio of the two sensors is RB/MW = 0.25/0.18~1.4. As this is not a large difference, we conclude that both sensors are able to detect the flexural mode frequencies of the concrete beam with good precision. It should be noted that the FFT scale is logarithmic and that is why the 1.4 factor discussed here is quite different than the 6.9 factor in Figure 5, where the vertical scale is linear.

### 4.2. Crack Detection by Sensors

Shown in Figure 10 is the crack detection experiment for the two sensors. The procedure is as follows. An undamaged concrete beam was introduced in the setup of Figure 7 and an FFT spectra was received (blue line in Figure 10) after the beam was excited, as described in the previous sections. After that, a single crack was introduced to the center of the beam and transverse to its length, on one side, and a new FFT spectrum was recorded in a similar fashion (orange line in Figure 10). To obtain an idea of the background baseline noise, an empty coil measurement was also performed (green line in Figure 10).

As there is a lot of noise in Figure 10 at frequencies below 5 kHz, this part of the spectrum was ignored (shaded area in the plots). It is clear from the two graphs that the appearance of the crack results in a negative shift of all three dominant peaks, as shown by the arrows and the dotted, vertical lines.

The peak values in Figure 10 are summarized in Table 4. Each sensor was attached to its own concrete beam, but the beam was the same before and after the crack insertion. Both samples showed three main peaks around approximately 5, 12, and 13 kHz. Shown in the table are the frequencies, f, of these peaks before damage, the same frequencies after a crack is introduced at the center of the beam, the corresponding change, Δf, and the relative change, Δf/f. This factor is the same for peaks 1 and 2 for both sensors, with corresponding values of 0.03 and 0.02, but is not exactly the same for the 3rd peak. The reason for this difference is not understood, but it should be noted that the concrete beams for the two sensors in this experiment are not the same and thus we did not expect identical results. In general, all peaks shift to the left with negative Δf values, and the relative change Δf/f is of the same order of magnitude between 0.01 and 0.03, proving that both sensors can be used for damage detection equivalently.

## 5. Comparison to Accelerometry Measurements

When using accelerometer measurements, as in [12,13,14,15,16,17], a modal analysis is performed by using Fast Fourier Analysis, similar to our technique. The damaged beam node frequencies are always lower than the corresponding healthy beam frequencies, which is expected as the cracks reduce the beam’s stiffness, and the node frequencies are proportional to the square root of stiffness. This behavior was also observed in the current study. In particular, the relative frequency shifts in [13] are of the order of 3 to 10% (Figure 5), in [14] reach a maximum of 10%, in [15] 1 to 5% (Table 3), and finally in [17] of the order of 10% (Table 4) for multiple cracks. These values are comparable to our result in the range of 1–3% in Table 4. Also, in [15], an impact hammer test was performed, similar to our dropping mass excitation. And, finally, in [16], carbon nanotubes are used as reinforcements in cement beams and as sensing elements, similar to our idea to perform the same activity with magnetic microwires.

It is hard to relate these frequency shifts to structural damage. There are a couple of good mechanical models, like [12,29], which take into account the beam stiffness, mass, and damping, and relate their properties (they are in the form of matrices) to the modal frequencies. All these models agree that the cracks introduce stiffness reduction (the stiffness is defined as the product EI, where E is the Young’s modulus of the material and I the moment of area of the structure, and as the bending frequencies depend on the square root of EI, a stiffness reduction leads to a decrease in the frequencies. However, this dependance is very complicated and non-linear in nature, as it depends on the distribution of the various wavelengths on the structure. For example, if damage occurs on a node of a standing wave, the corresponding frequency will have a zero shift, as there is no vibration locally on the node and thus the wave is not affected.

## 6. Conclusions

The behavior of magnetoelastic sensors for concrete beam damage detection was studied for two different sensor shapes: ribbon and microwire. Ribbons have been used successfully in the past for this purpose, but this was the first time microwires were used. The long-term plan is to embed them inside cement beams so as to act both as fiber reinforcements and as sensing material. In the current work, the sensors are placed only on the external surface of a cement beam, as the measurements are easier to make compared to the embedded case, in an effort to compare the two sensing materials and test the ability of the microwire to reproduce ribbon measurements.

The response of the two sensors received from a pick-up coil under an AC magnetic excitation showed that, in the case of the ribbon, the signal was stronger compared to the wire by a factor of 7. This can be explained by the difference in the cross-sectional areas of the two sensors and the difference in their magnetic properties. The thermal pre-annealing of the sensors led to stronger signal gain, in the range of 10 to 30%, with a slightly higher percentage for the ribbon than the microwire. On the other hand, the detected flexural modes of the concrete beams were literally not affected by the annealing process (changes less than 1%). Finally, a frequency nodal analysis comparison was made between the two sensors concerning damage detection. Both sensors detected a relative frequency shift of the order in the range of −1% to −3%, with almost identical results to each other, when a single crack was introduced to the concrete beams. This proves that both sensors produce almost equivalent results for the task of damage detection. As these sensors are contactless and the concrete material has no magnetic properties, they can be easily embedded inside the beams. The microwire shape is more advantageous for this task as the ribbon can lead to microlaminations and anisotropic local stresses, side effects that can be eliminated by the microwires due to their symmetric shape.

The current limitations of our future work are the signal attenuation at depths below a few millimeters, and the detection method, which, even though it is contactless, involves the encirclement of the beam by a coil. Both problems can be eliminated by proper signal filtering and amplification because stronger signals will make it possible to use coils vertically along the beam’s length and not place them around it, and the emitted signal can be made to originate from the inside of the beam. Another important limitation is the price of the microwires. Contrary to the ribbons that are produced on a large scale as they are basic transformer materials and they cost a few cents per meter, microwires are still produced individually in scientific labs; thus, their cost is significant. In order to use them as inclusion fibers inside concrete, their production cost must be reduced.

## Figures and Tables

**Table 1:** Magnetic properties of the two sensing materials.

	Saturation Magnetization Ms (T)	Saturation Magnetization Ms Literature (T)	Mangetostriction λ (ppm)	Magnetic Susceptibility χ
Ribbon sensor	0.9	0.9	12	57 × 10^4^
Microwire sensor	0.65 (at the maximum applied H)	1.6	32	4.4 × 10^4^

**Table 2:** Comparison of annealed versus non-annealed sensing data of Figure 8a,b (“a.u.”: arbitrary units).

	Ribbon Sensor	Annealed Ribbon	Difference %	Wire Sensor	Annealed Wire	Difference %
Peak 1 Amplitude (a.u.)	−5.73 × 10^4^	−4.97 × 104	15	−7.86 × 104	−7.17 × 104	9
Peak 1 Frequency (kHz)	5.59	5.59	0	5.67	5.65	0.3
Peak 2 Amplitude (a.u.)	−6.08 × 104	−4.59 × 104	32	−8.92 × 104	−7.18 × 104	24
Peak 2 Frequency (kHz)	12.5	12.5	0.25	12.7	12.6	0.14

**Table 3:** Percentage difference for the two sensors of the peak positions in the spectra of Figure 10.

Frequency (kHz)	Wire	Ribbon	Difference %
Peak 1	5.66	5.58	1.4
Peak 2	12.67	12.63	0.3
Peak 3	13.00	12.91	0.6

**Table 4:** Shift of mode frequencies due to the appearance of the crack for the two sensors in the spectra of Figure 10.

Frequency (kHz)		Ribbon	Wire
Peak 1	f no-crack	5.79	5.64
	f crack	5.61	5.49
	Δf	−0.18	−0.15
	Δf/f	−0.03	−0.03
Peak 2	f no-crack	11.44	12.66
	f crack	11.22	12.44
	Δf	−0.22	−0.22
	Δf/f	−0.02	−0.02
Peak 3	f no-crack	12.69	12.96
	f crack	12.3	12.83
	Δf	−0.39	−0.13
	Δf/f	−0.03	−0.01

## Data Availability

All data in the form of graphs are contained within the article. The raw data are available under request to the corresponding author.

## References

[1] Wu T., Liu G., Fu S., Xing F. (2020). Recent progress of fiber-optic sensors for the structural health monitoring of civil infrastructure. Sensors.

[2] Ono K. (2018). Review on structural health evaluation with acoustic emission. Appl. Sci..

[3] Varanis M., Silva A., Mereles A., Pederiva R. (2018). MEMS accelerometers for mechanical vibrations analysis: A comprehensive review with applications. J. Braz. Soc. Mech. Sci. Eng..

[4] Ceylan H., Gopalakrishnan K., Kim S., Taylor P.C., Prokudin M., Buss A.F. (2013). Highway infrastructure health monitoring using micro-electromechanical sensors and systems (MEMS). J. Civ. Eng. Manag..

[5] Han H.S., Duong T.A., Ahn C.W., Kim B.W., Lee J.S. (2023). A brief review on piezoelectrics-based paint sensors. J. Korean Inst. Electr. Electron. Mater. Eng..

[6] Khan Y., Thielens A., Muin S., Ting J., Baumbauer C., Arias A.C. (2020). A new frontier of printed electronics: Flexible hybrid electronics. Adv. Mater..

[7] Abbas M., Shafiee M. (2018). Structural health monitoring (SHM) and determination of surface defects in large metallic structures using ultrasonic guided waves. Sensors.

[8] Flah M., Nunez I., Ben Chaabene W., Nehdi M.L. (2021). Machine learning algorithms in civil structural health monitoring: A systematic review. Arch. Comput. Methods Eng..

[9] Avci O., Abdeljaber O., Kiranyaz S., Hussein M., Gabbouj M., Inman D.J. (2021). A review of vibration-based damage detection in civil structures: From traditional methods to Machine Learning and Deep Learning applications. Mech. Syst. Signal Process..

[10] Cao H., Liang N., Gu F. (2023). Vibration Structural Damage Diagnosis Based on Modal Parameters. Proceedings of the UNIfied Conference of DAMAS, IncoME and TEPEN Conferences (UNIfied 2023), Huddersfield, UK, 29 August–1 September 2023.

[11] Shi Z.Y., Law S.S., Zhang L.M. (2000). Structural damage detection from modal strain energy change. J. Eng. Mech..

[12] Hussein H.A., Rahim S.B.A., Mustapha F.B., Krishnan P.S., Jalil N.A.B.A. (2025). Data-driven multi-fault detection in pipelines utilizing frequency response function and artificial neural networks. J. Pipeline Sci. Eng..

[13] Aranizadeh A., Shad H., Vahidi B., Khorsandi A. (2025). A novel small-scale wind-turbine blade failure detection according to monitored-data. Results Eng..

[14] Hamza M., Akhtar K., Khan M.A. (2024). Modal and dynamic analysis of damaged steel column-beam frame structure subjected to seismic vibrations using experimental and finite element analysis approach. J. Braz. Soc. Mech. Sci. Eng..

[15] Ramakrishna S., Sathish J., Raj Kumar V., Raghu Vamsi S. (2022). Experimental investigation on crack localization in steel and composite structures by intersection of first three normalized mode shape curves. J. Fail. Anal. Prev..

[16] Rao R.K., Sasmal S. (2020). Smart nano-engineered cementitious composite sensors for vibration-based health monitoring of large structures. Sens. Actuators A Phys..

[17] Modesti M., Gentilini C., Palermo A., Reynders E., Lombaert G. (2024). A two-step procedure for damage detection in beam structures with incomplete mode shapes. J. Civ. Struct. Health Monit..

[18] Modzelewski C., Savage H., Kabacoff L., Clark A. (1981). Magnetomechanical coupling and permeability in transversely annealed Metglas 2605 alloys. IEEE Trans. Magn..

[19] Savage H., Spano M. (1982). Theory and application of highly magnetoelastic Metglas 2605SC. J. Appl. Phys..

[20] Song H., Jang Y., Lee J.P., Choe J.K., Yun M., Baek Y.K., Kim J. (2023). Highly compressible 3D-printed soft magnetoelastic sensors for human–machine interfaces. ACS Appl. Mater. Interfaces.

[21] Shekhar S., Karipott S.S., Guldberg R.E., Ong K.G. (2021). Magnetoelastic sensors for real-time tracking of cell growth. Biotechnol. Bioeng..

[22] Samourgkanidis G., Kouzoudis D. (2020). Characterization of magnetoelastic ribbons as vibration sensors based on the measured natural frequencies of a cantilever beam. Sens. Actuators A Phys..

[23] Vázquez M. (2007). Advanced magnetic microwires. Handbook of Magnetism and Advanced Magnetic Materials.

[24] Zhukov A., Ipatov M., Corte-León P., Gonzalez-Legarreta L., Blanco J., Zhukova V. (2020). Soft magnetic microwires for sensor applications. J. Magn. Magn. Mater..

[25] Panina L., Makhnovskiy D., Morchenko A., Kostishin V. (2015). Tunable permeability of magnetic wires at microwaves. J. Magn. Magn. Mater..

[26] Salaheldeen M., Garcia-Gomez A., Corte-León P., Gonzalez A., Ipatov M., Zhukova V., Gonzalez J., Antón R.L., Zhukov A. (2023). Manipulation of magnetic and structure properties of Ni2FeSi glass-coated microwires by annealing. J. Alloys Compd..

[27] Tapeinos C.I., Kamitsou M.D., Dassios K.G., Kouzoudis D., Christogerou A., Samourgkanidis G. (2023). Contactless and Vibration-Based Damage Detection in Rectangular Cement Beams Using Magnetoelastic Ribbon Sensors. Sensors.

[28] (2005). Methods of Testing Cement–Part 1: Determination of Strength.

[29] Xiao F., Mao Y., Tian G., Chen G.S. (2024). Partial-Model-Based Damage Identification of Long-Span Steel Truss Bridge Based on Stiffness Separation Method. Struct. Control. Health Monit..

